# Effects of muscle shortening on single-fiber, motor unit, and compound muscle action potentials

**DOI:** 10.1007/s11517-021-02482-z

**Published:** 2021-12-22

**Authors:** Javier Rodriguez-Falces, Armando Malanda, Javier Navallas

**Affiliations:** Department of Electrical and Electronic Engineering. Public, University of Navarra, Campus de Arrosadía s/n., 31006 Pamplona, Spain

**Keywords:** Muscle shortening, Muscle architecture, EMG modeling, Surface motor unit potential, Isometric contraction, End-of-fiber components, Non-propagating components

## Abstract

Even under isometric conditions, muscle contractions are associated with some degree of fiber shortening. The effects of muscle shortening on extracellular electromyographic potentials have not been characterized in detail. Moreover, the anatomical, biophysical, and detection factors influencing the muscle-shortening effects have been neither identified nor understood completely. Herein, we investigated the effects of muscle shortening on the amplitude and duration characteristics of single-fiber, motor unit, and compound muscle action potentials. We found that, at the single-fiber level, two main factors influenced the muscle-shortening effects: (1) the electrode position and distance relative to the myotendinous zone and (2) the electrode distance to the maxima of the dipole field arising from the stationary dipole created at the fiber-tendon junction. Besides, at the motor unit and muscle level, two additional factors were involved: (3) the overlapping between the propagating component of some fibers with the non-propagating component of other fibers and (4) the spatial spreading of the fiber-tendon junctions. The muscle-shortening effects depend critically on the electrode longitudinal distance to the myotendinous zone. When the electrode was placed far from the myotendinous zone, muscle shortening resulted in an enlargement and narrowing of the final (negative) phase of the potential, and this enlargement became less pronounced as the electrode approached the fiber endings. For electrode locations close to the myotendinous zone, muscle shortening caused a depression of both the main (positive) and final (negative) phases of the potential. Beyond the myotendinous zone, muscle shortening led to a decrease of the final (positive) phase. The present results provide reference information that will help to identify changes in MUPs and M waves due to muscle shortening, and thus to differentiate these changes from those caused by muscle fatigue.

**Graphical abstract:**

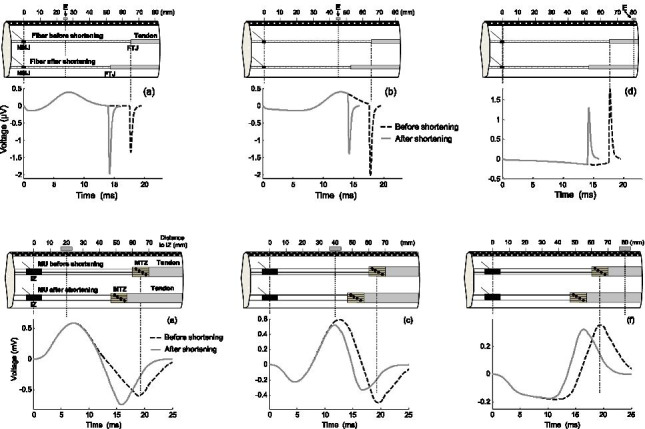

## Introduction

During voluntary isometric contractions, muscles undergo important changes in their architecture, such as shortening of fascicle lengths [22, 26], increases in pennation angles [20], fascicle rotation [5], and alterations in the relative orientation of muscle fibers with respect to the detection system [10]. Among the above architectural alterations, muscle shortening is particularly relevant due to its mechanical, physiological, and electrical implications. In mechanical terms, shortening of fascicle lengths during an isometric contraction is the primary cause for the increase in the physiological cross-sectional area, a parameter related to force production capacity [17]. With regard to physiological implications, fiber shortening is accompanied by an increase in the diameter of individual muscle fibers, which augments the conduction velocity of action potentials along the fibers [15, 28].

In electrical terms, a reduction of the fiber length causes alterations in the shape of individual single-fiber action potentials (SFAPs) and also in the pattern of summation of these SFAPs in motor unit potentials (MUPs) and compound muscle action potentials (M waves). However, previous studies on muscle shortening have concentrated more on assessing how such shortening influences the spectral and amplitude characteristics of the interference surface EMG signal [24, 36], rather than on examining the effects on the shape of individual SFAPs, MUPs, and M waves. A detailed characterization of the muscle-shortening effects will help to identify the changes in experimental MUPs and M waves caused by muscle shortening, and thus to distinguish these changes from those caused by muscle fatigue (reduction in conduction velocity, elongation of the intracellular action potential).

In a previous work of our group, the effects of muscle shortening were investigated on individual motor unit potentials detected under bipolar configuration [31]. Unfortunately, under bipolar configuration, part of the electrical signal is lost due to phase cancelation [32]. To overcome this limitation, in the present study, a monopolar electrode configuration was adopted since monopolar signals contain the genuine electrical content associated to the action potential propagation and extinction, thus allowing the analysis of the muscle-shortening effects in their full extent and authenticity. Besides, in our previous study, muscle-shortening effects were examined only on MUPs [31]: the present study extends this line of investigation to M waves and SFAPs.

**Fig. 1:**
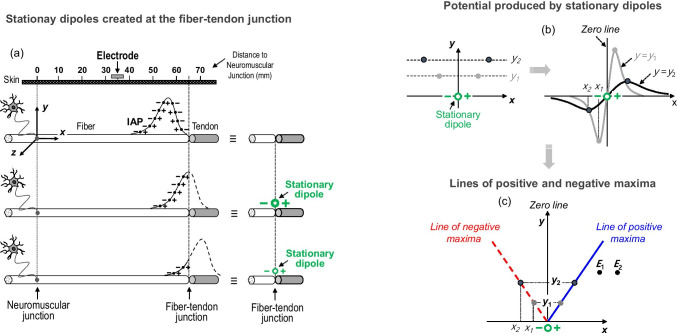
(a) Schematic representation of the process of extinction of the IAP source at the fiber-tendon junction. On the right, the stationary dipoles created at the junction during the IAP extinction are shown. (b) Dipole potentials recorded at a short (*y*_1_) and a long (*y*_2_) radial distance. (c) Lines containing the positions of the positive maxima and negative maxima of the dipole potentials at the two radial distances indicated in (b)

There are several factors influencing SFAP, MUAP, and M-wave characteristics during muscle shortening. To understand these factors, one needs first to understand that extracellular potentials are formed by two different constituents: a propagating component (generated by the propagation of the action potential along the muscle fibers) and a non-propagating, end-of-fiber, component (originated by the extinction of the action potential at the myotendinous junctions). When the fiber is shortened, the distance from the myotendinous zone to the recording electrode is modified, which affects the characteristics of the end-of-fiber component: thus, it appears that detection factors (such as the electrode relative position) would influence the shortening effects. In addition, it is known that a stationary dipole is created at the fiber-tendon junctions (see Fig. 1 of the present paper and also [23]). Hence, the electrode position relative to the maxima of the dipole field arising from the stationary dipole may also be involved. Finally, anatomical factors, such as the spreading length of the myotendinous zone, could also play a role in the shortening effects.

Investigating the muscle-shortening effects using an experimental approach is difficult. An alternative strategy is to use computational models, as they afford the possibility of studying the effect of muscle architectural parameters on the M-wave characteristics in isolation (or in combination) to changes in physiological variables. Besides, the use of simulations also permits to assess, in a systematic manner, the influence of the distance from the electrode to the myotendinous zone on the muscle-shortening effects.

The objective of the present study was to assess systematically the effects of muscle shortening on the amplitude and duration characteristics of individual SFAPs, MUPs, and M waves for different positions of the electrode along the muscle longitudinal axis. To obtain a more comprehensive understanding of the shortening effects, we proposed two models of extracellular EMG potentials; one analytical, which enables visualization of the changes in the shape of potentials, and another biophysical, which facilitates understanding of the factors involved in muscle shortening. The study was focused solely on the impact of changes in the geometric aspects of the muscle; i.e., we assumed that no physiological alterations accompanied fiber shortening.

## Material and methods

The study included two different approaches to examine and understand the consequences of muscle shortening on EMG potentials. The first method consisted of a biophysical description of the electrical field that emerges from the stationary dipole formed at the fiber-tendon junction when the intracellular action potential (IAP) reaches the fiber ends [30]. This biophysical model had the advantage of offering a simple schematic representation of the end-of-fiber potentials, thereby facilitating the understanding of the fiber-shortening effects.

The second method involved computer simulations based on an analytical model of single-fiber potentials described previously [9]. This model allowed us to synthesize SFAPs, MUPs, and M waves with the known physiological properties of the *biceps brachii* muscle [11]. This model was constructed on the basis of an analytical description of the IAP, and, therefore, enabled a detailed assessment of the changes in the shape of extracellular potentials caused by muscle shortening.

### Biophysical model of end-of-fiber potentials

*End-of-fiber* signals are potentials that arise when the intracellular action potentials reach the ends of the muscle fibers. *End-of-fiber* signals play a key role in the muscle-shortening effects. The reason is that, when the fiber is shortened, the distance from the myotendinous zone to the recording electrode changes, which has a direct impact in the end-of-fiber component. Therefore, to fully understand the muscle-shortening effects, we present a biophysical model for the *end-of-fiber* potentials, which allows easy visualization of the changes in the amplitude of these potentials resulting from positional changes of the myotendinous junction relative to the recording electrode.

#### Stationary dipoles created at the fiber-tendon junction

An electric dipole consists of two point current sources of opposite polarity separated by a small distance. In the context of bioelectricity, the dipole can be considered the elementary electrical source. Indeed, the IAP can be modeled as a collection of infinite lumped dipoles distributed along its spatial profile [33], as shown in Fig. 1(a). When the IAP reaches the end of the fiber, it does not disappear abruptly; rather, it vanished gradually. During the process of extinction of the IAP, there is a gradual change in the dipole moment of the whole IAP source. This change in the dipole moment is due to progressive vanishing of the distributed dipoles of the IAP profile that should have existed beyond the fiber-tendon junction had the fiber been infinite (Fig. 1(a)) [2]. As a result, during the process of IAP extinction, the net electrical source can be approximated as a stationary (dipole) source of changing intensity [23].

#### Extracellular potential generated by stationary dipoles: lines of positive and negative maxima

The stationary dipole created at the fiber-tendon junction generates a potential field with non-propagating or “standing” character. The potential produced by a stationary dipole along a line located at a certain radial distance (*y*_1_, *y*_2_) from the dipole axis is a biphasic (negative–positive) waveform, as shown in Fig. 1(b). This potential is zero at the perpendicular “zero line” shown in Fig. 1(b). It is important to note that the dipole potential becomes broader as the radial distance increases: for example, note how the maximum of the potential gets further from the zero line as radial distance increases from *y*_1_ to *y*_2_. In a previous study, we derived the mathematical expression that relates the position (in the *x*-axis) of the maximum of a potential as a function of the radial distance (*y*_i_) at which this potential is recorded [30]:

1 $$x=\pm \frac{y}{\sqrt{2}}\approx \pm 0.7 y$$

Equation (1) provides the analytical expression of the straight lines that contain the (x, y) coordinates of the positive and negative peak points of the dipole potential at different radial distances (Fig. 1(c)). The lines of positive and negative maxima offered a direct straightforward representation of the potential produced by a dipole in the extracellular medium. With this representation, it can be seen that, for a fixed radial distance, the closer the electrode to the lines of positive or negative maxima, the greater the potential (Fig. 1(c)). As an example, the potential at electrode 1 (*E*_1_) would be larger than at electrode 2 (*E*_2_) because *E*_1_ is closer to the line of positive maxima emerging from the dipole.

### Analytical model of extracellular EMG potentials

Extracellular potentials produced by individual muscle fibers were calculated according to the model proposed by Dimitrov and Dimitrova (1998) [9]. In this model, each individual muscle fiber was considered a time-shift invariant system, and each SFAP was computed as the convolution of the input signal and the impulse response (IR) of the corresponding system.

2 $$SFAP\left(t\right)={C}_{an}\bullet \frac{\partial IAP\left(t\right)}{\partial t}*IR (t)$$

In Eq. (2), the input signal was the first temporal derivative of the IAP. In the present study, we used the IAP analytical expression defined in Rodriguez-Falces et al. (2012) [29], which is a slight modification of the original model proposed by Dimitrov and Dimitrova (1998) [9]. This description allows a fine control of the IAP shape. Importantly, we made the duration of the IAP spike to be 1.0 ms, in agreement with recent findings [29]. The impulse response in Eq. (2) represented the sum of the potentials generated at the observation point by two dipoles propagating in opposite directions from the innervation zone to the fiber ends. This impulse response included (1) the properties of each muscle fiber, such as the length of the fiber and its conduction velocity; (2) the detection conditions, such as the radial distance and the longitudinal distance of the electrode relative to the end-plate; (3) and the properties of the volume conductor (infinite, homogeneous, and anisotropic, with the conductivity in the longitudinal fiber direction five times higher than in transverse direction). Details of such impulse response can be found in [31].

Assuming that the physiological properties of the fiber do not change from one fiber to another, the IAP in time domain could be accepted as identical for all fibers of the motor unit, regardless of their diameters [12]. Then, the motor unit can also be considered a linear time shift-invariant system, whose common impulse response is the sum of *N* impulse responses corresponding to individual muscle fibers [12]. Instead of N convolutions (one for every fiber), the motor unit potential (MUP), as the output signal, can be calculated as a single convolution as follows:

3 $$MUP\left(t\right)={C}_{an}\bullet \frac{\partial IAP\left(t\right)}{\partial t}*\sum_{i=1}^{i=N}{IR}_{i}(t)$$

The anatomical and physiological properties of the simulated muscle fibers and their organization within the muscle were chosen so to mimic those of the *biceps brachii* muscle [21]. First, in each motor unit, muscle fibers were assumed to run parallel to the muscle longitudinal axis. The average semi-length of the fibers was 65 mm. For each motor unit, the average fiber diameter was 55 µm and varied within a range of 10 µm. The innervation points were scattered, with normal distribution, within ± 2 mm around the point that divided the fibers into two portions of equal length. For each motor unit, we assumed that both proximal and distal ends of the fibers were scattered, following a normal distribution, within ± 5 mm around the mean position of the corresponding end. Thus, the default value used for the spreading length of the myotendinous zone of each motor unit was 10 mm. Of note, the spreading of the myotendinous region was a design parameter that was allowed to change in the present simulation experiments.

M waves were simulated by summing the motor unit potentials generated by the motor units in the muscle. The simulated muscle had a circular cross-section with a diameter of 28 mm, in accordance with measures of physiological cross-sectional areas of the *biceps brachii* [39]. The muscle comprised a total of 700 motor units [8]. The distributions of properties of the motor units (i.e., innervation number, territory, and conduction velocity) were based on the size principle [16]. Accordingly, the territories of the largest motor units were greater than those of the smallest motor units [6]. Motor unit territories were randomly distributed throughout the muscle [18, 25]. The fibers of a motor unit were randomly scattered in the motor unit territory [38] with a density of 20 fibers/mm^2^ [3] and intermingled with fibers belonging to other units. The conduction velocity of the 700 motor units was spread following a Gaussian distribution [14] with the smallest motor units assigned the slowest conduction velocities [1]. The mean value of motor unit conduction velocity was 3.7 ± 0.5 m/s, in agreement with the direct measurements in individual muscle fibers [37].

The simulated signals were detected using monopolar configuration. This electrode arrangement enables recording of the entire informative content resulting from the IAP propagation and extinction, which means that there is no signal cancelation as in bipolar configuration [32]. Thus, theoretically, under monopolar configuration, the muscle-shortening effects are expressed in their full authenticity, as these signals are not contaminated or blurred by the signal cancelation problem. It is important to note that the monopolar configuration cannot be considered similar or equivalent to the belly-tendon configuration (an electrode arrangement often adopted in the *biceps brachii*) as the “tendon” electrode may record far-field potentials of significant amplitude: for example, the tendon electrode could pick up end-of-fiber potentials from the active muscle [19]. The electrodes were circular, with a diameter of 8 mm. The EMG signals were simulated using a sampling frequency of 5 kHz.

#### Simulation of changes in muscle architecture during shortening

**Fig. 2:**
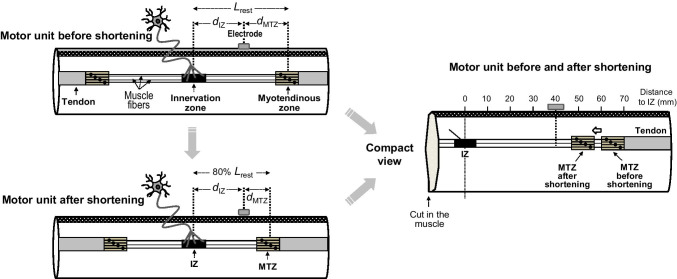
Left panel—schematic representation of a motor unit before (top) and after (bottom) reducing the fibers’ semi-length at rest (Lrest) by 20%. Note that, when the motor unit is shortened, the proximal and distal myotendinous zones (MTZs) are moved closer to the innervation zone (IZ). Thus, muscle-shortening results in a decrease of the distance between the electrode and myotendionous zone (*d*_MTZ_), but in no change in the distance between electrode and innervation zone (*d*_IZ_). Right panel—Compact view of the right semi-half of the muscle, which includes a motor unit in both resting and shortened conditions. For sake of clarity, the resting and shortened motor units have been drawn at different depths in the muscle to better visualize the shortening phenomenon

For all simulations, it was assumed that the muscle was contracted isometrically. During the isometric contraction, each fiber was shortened by the two ends being moved closer to the neuromuscular junction (Fig. 2). For simplicity, we considered that each muscle fiber was shortened symmetrically about the neuromuscular junction, which lay approximately at the center of muscle fibers (see above). This means that, when the muscle was shortened, the distance between the electrode and the myotendinous zone (*d*_MTZ_) was altered compared to the resting muscle, while the distance from the electrode to the innervation zone (*d*_IZ_) remained unchanged (Fig. 2). As defined above, the mean fiber semi-length at rest was 65 mm (from now on referred to as Lrest) and the muscle was shortened by reducing the fibers’ length by up to 20% of Lrest, in agreement with the degrees of muscle shortening found during in vivo isometric contractions [22, 26]. Muscle shortening was considered to have no impact on fiber membrane properties. There was no shift of the skin relative to the muscle.

#### Nomenclature for extracellular EMG potentials depending on the electrode longitudinal position

**Fig. 3:**
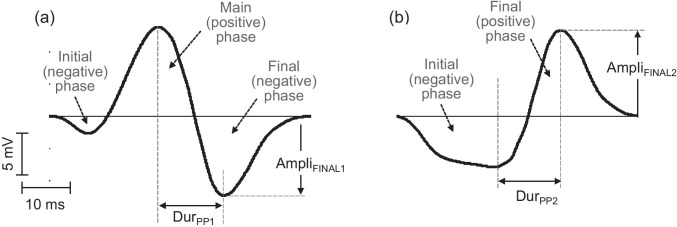
Representative examples of surface-detected potentials simulated between the innervation and myotendinous zones (**a**) and beyond the myotendinous zone (**b**). The different phases and parameters of each potential are indicated

A general notation is described next to standardize the nomenclature used throughout the study. In surface EMG, when the electrode lies close to the innervation zone, extracellular potentials normally have a biphasic (positive–negative) waveform. However, as the electrode is moved further from the innervation zone, an initial small (negative) phase eventually appears preceding the two principal phases, thereby transforming the potential into a triphasic waveform (Fig. 3a). This initial small phase was disregarded in the present study and we focused our attention solely on the posterior phases, as shown in Fig. 3. Two parameters were defined for this waveform: the time interval between the main (positive) peak and the final (negative) peak (*Dur*_PP1_), and the amplitude of the final (negative) phase (*Ampli*_FINAL1_). For electrode locations beyond the myotendinous zone, extracellular potentials normally have a biphasic (negative–positive) waveform (Fig. 3b). For these beyond-the-tendon potentials, two parameters were measured: the time interval between the initial (negative) peak and the final (positive) peak (*Dur*_PP2_), and the amplitude of the final (positive) phase (*Ampli*_FINAL2_).

#### Simulated conditions

Several sets of simulations were performed to characterize the effects of muscle shortening on the shape features of single-fiber and compound extracellular potentials. Since the waveform characteristics of surface-detected potentials are critically dependent on the electrode position (see Fig. 3), the muscle-shortening effects were examined for different locations of the electrode relative to the myotendinous zone. At the single-fiber, motor unit, and muscle levels, comparisons were made between potentials simulated at two conditions: one for the muscle at rest, and the other for a muscle whose length was reduced by 20%. Other simulation details and experiments performed are the following.

In the case of single-fiber potentials, the shortening effects were analyzed using both the analytical and biophysical models. In these simulations, the distance from the muscle fiber to the recording electrode (the so-called radial distance) was 15 mm.

To characterize the effects of muscle shortening on the shape of motor unit potentials, a motor unit located at a radial distance of 15 mm from the electrode was chosen as a reference, and the shortening effects were assessed for this particular motor unit for different positions of the electrode relative to the myotendinous region and also for two different spreading lengths (narrow and wide) of the myotendinous region. In addition, to investigate the muscle-shortening effects in the whole population of motor units in the muscle, the average changes (mean ± SD) in MUP characteristics (*Ampli*_*FINAL1*_, *Ampli*_*FINAL2*_, *Dur*_*PP1*_, and *Dur*_*PP2*_) caused by muscle shortening for different positions of the electrode along the MU longitudinal axis were calculated from all motor units in the simulated muscle.

For compound potentials (MUPs and M waves), the spreading length of the myotendinous region directly affects the pattern of summation of the end-of-fiber components corresponding to the different fibers of a motor unit/muscle. Thus, this parameter has a great impact on the electrical formation of MUPs and M waves. Therefore, in these composite potentials, muscle-shortening effects were examined for two different spatial lengths of the myotendinous region of the motor unit: one narrow (range = 10 mm) and the other wide (range = 20 mm). Moreover, in the case of M waves, additional scenarios were simulated in which motor units were scattered over wider regions (range = 30 and 40 mm). These scenarios fit well with the real geometry of the *biceps brachii*, as the distal biceps tendon has been found to flatten into a straplike internal aponeurosis whose length is roughly 30% of the biceps length [27].

## Results

### Muscle-shortening effects for SFAPs

**Fig. 4:**
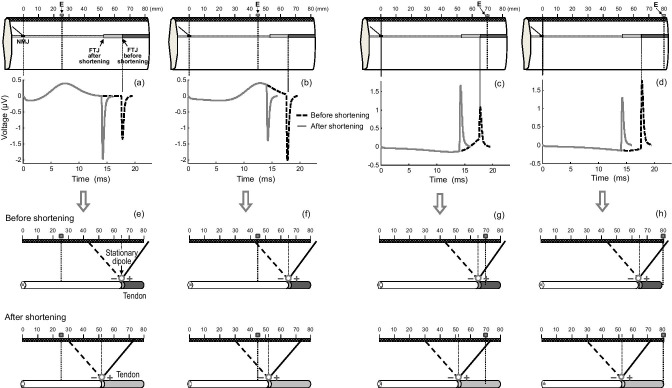
Upper panel—(a–d) simulation of changes in the SFAP waveform produced by a fiber shortening of 20% at 4 different positions of the point electrode (E) along the muscle longitudinal axis. Bottom panel—(e–h) schematic representation of the lines of positive and negative maxima emerging from the stationary dipoles that arise at the fiber-tendon junction (FTJ) due to the action potential extinction. Radial distance was 15 mm

The first step was to describe the fiber-shortening effects in individual muscle fibers, as this allowed the study of the shortening effects without the confounding influence of the phase summation/cancelation present in compound potentials. Figure 4 shows the changes in the SFAP waveform caused by fiber shortening at 4 different electrode locations along the muscle longitudinal axis. Below each of the SFAPs, a schematic representation of the lines of positive and negative maxima emerging from the stationary dipoles is shown. It can be seen that fiber-shortening effects changed dramatically depending on the electrode longitudinal position. For clarity, the results were reported in two separate sections based on the electrode position relative to the fiber-tendon junction.

In the first section, electrode placed between the neuromuscular and fiber-tendon junctions, it can be seen that, for electrode locations far from the tendon junction (*d*_IZ_ = 25 mm, Fig. 4(a)), fiber shortening caused an increase in the SFAP final phase, whereas the opposite occurred for electrode locations close to this junction (*d*_IZ_ = 45 mm, Fig. 4(b)). At this point, inspection of the lines of positive and negative maxima revealed that, for the scenario with the electrode at *d*_IZ_ = 25 mm, fiber shortening moved the line of negative maxima closer to the electrode (Fig. 4(e)), while, for the electrode at *d*_IZ_ = 45 mm, the line of negative maxima ended up further from the electrode after shortening (Fig. 4(f)).

In the second section, electrode placed beyond the fiber-tendon junction, it can be seen that, for electrode locations a little beyond tendon junction (*d*_IZ_ = 70 mm, Fig. 4(c)), fiber shortening caused an increase in the SFAP final phase, whereas, for electrode locations far beyond this junction (*d*_IZ_ = 80 mm, Fig. 4(d)), the SFAP final phase decreased when the fiber was shortened. Again, inspection of the lines of positive and negative maxima helps us understand these results. Indeed, it can be seen that, for the electrode at *d*_IZ_ = 70 mm, fiber shortening brought the line of positive maxima closer to the electrode (Fig. 4(g))], while, for the electrode at *d*_IZ_ = 80 mm, the line of positive maxima ended up further from the electrode after shortening (Fig. 4(h)).

### Muscle-shortening effects for MUPs

#### Influence of electrode longitudinal position on muscle-shortening effects

**Fig. 5:**
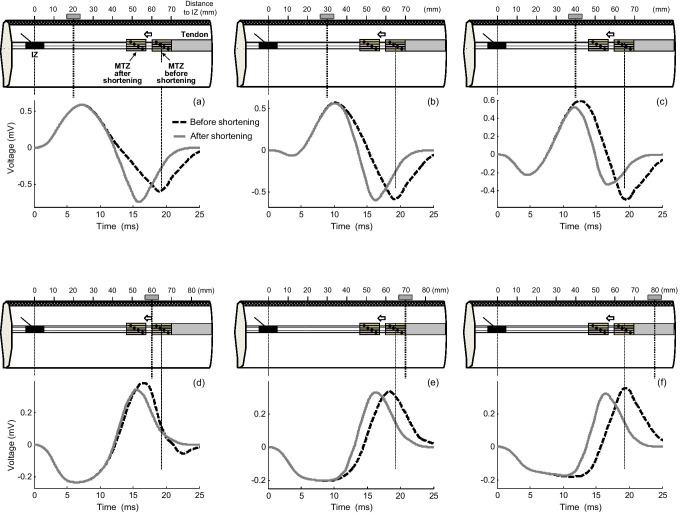
Simulation of changes in the MUP waveform produced by a shortening of the motor unit fibers of 20% at various positions of the electrode between the innervation zone (IZ) and myotendinous zone (MTZ) (upper panel—(a–c)) and at various electrode locations beyond the myotendinous zone (bottom panel—(d–f)). Radial distance was 15 mm

Again, for clarity in the exposition, the shortening effects on MUPs were reported in two separate sections. In the first section (electrode placed between the innervation and myotendinous zones, upper panel of Fig. 5), it can be seen that, when the electrode was far from the fiber endings (*d*_IZ_ = 20 mm, Fig. 5(a)), muscle shortening resulted in a marked enlargement and narrowing of the MUP final (negative) phase. This enlargement and narrowing of the final phase was less pronounced as the electrode approached the myotendinous zone (for example at *d*_IZ_ = 30 mm, Fig. 5(b)). Moreover, for electrode locations close to the fiber endings (*d*_IZ_ = 40 mm, Fig. 5(c)), muscle shortening caused a decrease in the amplitude of final negative phase (also in the amplitude of positive phase) of the MUP.

When the recording electrode was placed above or beyond the myotendinous region (lower panel of Fig. 5), muscle shortening led to a decrease of the final (positive) phase of the beyond-the-muscle MUP. The extent of this decrease depended on the specific position of the electrode (see next paragraph). Additionally, irrespective of the electrode position and distance to the myotendinous zone, two additional observations were made: first, muscle shortening resulted in a reduction of the MUP duration, and second, the changes in the amplitude of the MUP phases caused by shortening were much less pronounced than those of the SFAP phases.

**Fig. 6:**
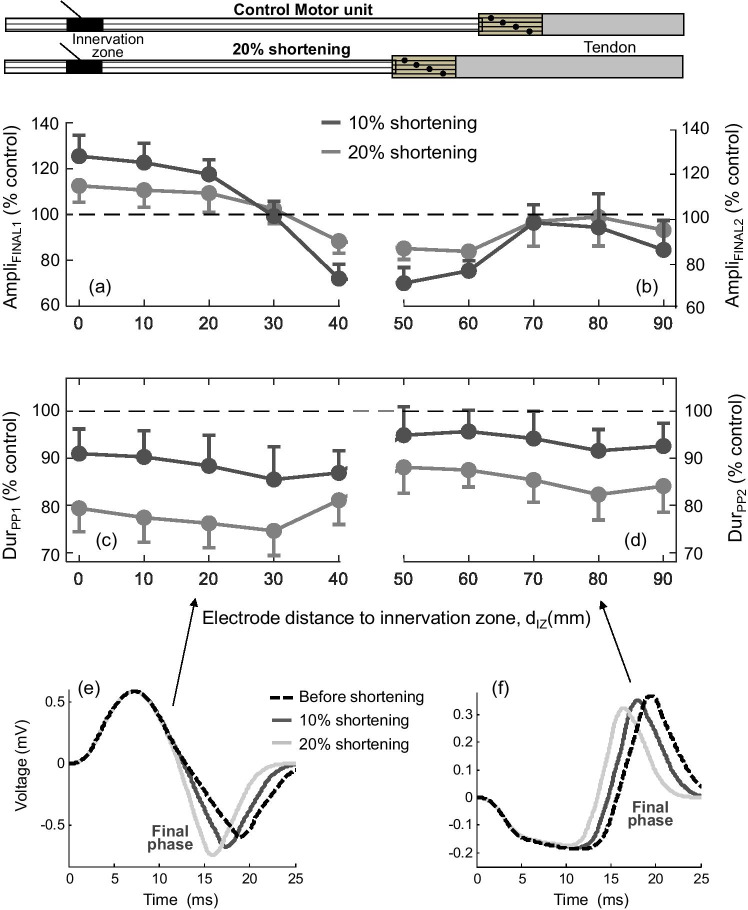
Simulation of average changes (mean ± SD) in MUP parameters, Ampli_FINAL1_ (a), Ampli_FINAL2_ (b), Dur_PP1_ (c), and Dur_PP2_ (d) produced by muscle shortenings of 10 and 20% relative to the MUP simulated at resting length as a function of the distance from the electrode to the innervation zone (*d*_IZ_). At the bottom, two series of superimposed MUPs simulated at longitudinal distances of *d*_IZ_ = 20 mm (e) and 80 mm (f), each series containing various MUPs obtained for different degrees of muscle shortening

Figure 6 shows the average changes in MUP characteristics (Ampli_FINAL1_ (a), Ampli_FINAL2_ (b), Dur_PP1_ (c), and Dur_PP2_ (d)) produced by different degrees of muscle shortening for different positions of the electrode along the MU longitudinal axis, obtained from all motor units in the simulated muscle. It can be seen that the enlargement of MUP final phase (Ampli_FINAL1_) induced by muscle shortening was maximal over the innervation zone and that this enlargement was gradually attenuated as the electrode approached the myotendinous region (Fig. 6(a)). Indeed, the increase in Ampli_FINAL1_ was reversed into a decrease at *d*_IZ_ = 30 and 40 mm. On the other hand, muscle shortening caused a decrease of the final phase of the beyond-the-muscle MUP (Ampli_FINAL2_) for all positions above and beyond the muscle (Fig. 6(b)). Importantly, this decrease was more pronounced above (*d*_IZ_ = 50, 60 mm) and far beyond (*d*_IZ_ = 80, 90 mm) the myotendinous region than a little beyond this zone (*d*_IZ_ = 70 mm).

Figure 6(c, d) shows that muscle shortening led to a narrowing of the MUP irrespective of the electrode longitudinal position. The reduction in MUP duration was most pronounced far from the tendon junction (*d*_IZ_ between 0 and 40 mm) and far beyond this junction (*d*_IZ_ = 80 and 90 mm), but this reduction was attenuated above the myotendinous region (*d*_IZ_ = 50, 60, and 70 mm).

#### Influence of the length of the myotendinous region on muscle-shortening effects

**Fig. 7:**
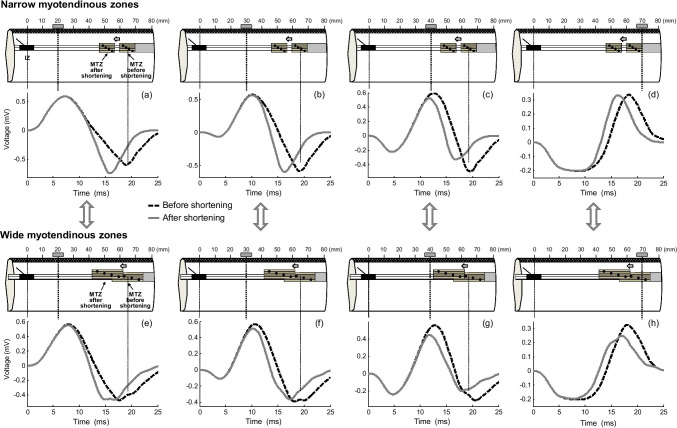
Comparison of the changes in the MUP waveform produced by a muscle shortening of 20% between a motor unit with a narrow myotendinous zone (range = 10 mm, upper panel) and a motor unit with a wide myotendinous zone (range = 20 mm, bottom panel) for various electrode locations along the muscle. Radial distance was 15 mm

The effects of muscle shortening were examined for two different spreading lengths of the myotendinous region of the motor unit: one narrow (range = 10 mm) and the other wide (20 mm) (see top and bottom rows, respectively, of Fig. 7). It can be seen that an increase in the spread of the fiber endings caused significant alterations in the shortening effects. First, a widening of the myotendinous region reduced the increasing effect of muscle shortening on the MUP final phase. For example, for the electrode at *d*_IZ_ = 20 mm, muscle shortening induced an enlargement of the MUP final phase in a motor unit with a narrow myotendinous zone (Fig. 7(a)), but this increase was completely suppressed in a motor unit with a wide myotendinous zone (Fig. 7(e)). Also noteworthy is that a widening of the myotendinous zone accentuated the depressing effect of muscle shortening on the MUP main (positive) phase. As an example, for the electrode at *d*_IZ_ = 30 mm, the amplitude of the MUP main positive phase did not change due to muscle shortening for a narrow spreading of the fiber endings (Fig. 7(b)), whereas this amplitude was decreased by muscle shortening in the case of a wide spreading (Fig. 7(f)).

### Muscle-shortening effects for M waves

**Fig. 8:**
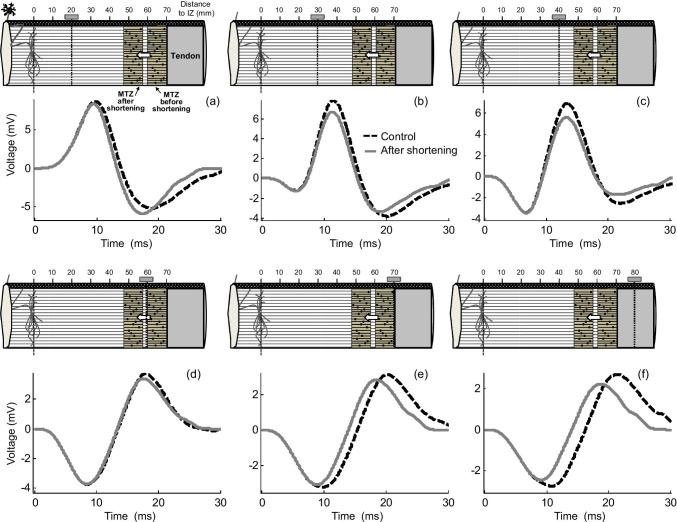
Simulation of changes in the M-wave waveform produced by a muscle shortening of 20% at various positions of the electrode between the innervation zone (IZ) and myotendinous zone (MTZ) (upper panel—(a–c)) and at various electrode locations beyond the myotendinous zone (bottom panel—(d–f))

In essence, the shortening effects on M waves were similar, although more attenuated, than those previously described for MUPs. Indeed, muscle shortening caused an enlargement of the M-wave final phase for electrode locations far from the tendon (Fig. 8(a)), but this enlargement was neutralized and even reversed into a depression as the electrode approached the tendon junction (Fig. 8(b, c)). Additionally, for electrode locations beyond the myotendinous region, muscle shortening caused a decrease of the M-wave final (positive) phase (low panel of Fig. 8). Besides, irrespective of the electrode position and distance to the tendon, a reduction of muscle length resulted in shorter M waves. Note that the changes in the magnitude and duration of the M waves provoked by shortening were less pronounced than those observed in MUPs.

#### Influence of the length of the myotendinous region on muscle-shortening effects

**Fig. 9:**
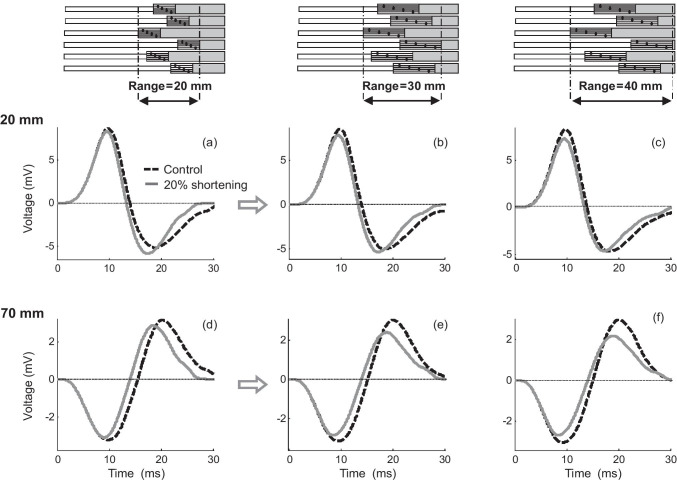
Comparison of the changes in the M-wave waveform produced by a muscle shortening of 20% for a muscle whose motor units have myotendinous regions with a default width (first column), a muscle whose motor units have wider myotendinous regions (second column), and a muscle whose wider myotendinous regions are scattered in a wider region (second column). The above comparisons are made for two electrode distances to the innervation zone: 20 and 70 mm

Figure 9 shows a comparison of the muscle-shortening effects on M waves for three muscles: (1) one whose motor units had myotendinous regions with normal widths (default muscle, first column), (2) another whose motor units had wider myotendinous regions (second column), and (3) another one whose motor units had wider myotendinous regions which, in addition, were scattered in a wider region (third column). It can be seen that, for electrode locations far from the tendon (*d*_IZ_ = 20 mm, first row), the enlargement of the M-wave final phase observed in the default muscle (+ 14%, Fig. 9(a)) was moderately attenuated in the muscle whose motor units had wider myotendinous regions (+ 8%, Fig. 9(b)), and completely supressed in the muscle whose myotendinous zones were scattered in a wider region (0%, Fig. 9(c)). Also, for electrode locations above and beyond the fiber endings (*d*_IZ_ = 70 mm, second row), we noted that muscle shortening induced a decrease of the M-wave final (positive) phase in the default muscle (+ 12%, Fig. 9(d)) and this depression was accentuated in muscles with wider myotendinous regions (+ 25 and 36% in the second and third simulated muscles, respectively, Fig. 9(e, f)).

## Discussion

The main finding of the present study was that the effects of muscle shortening on single-fiber, motor unit, and compound muscle action potentials depend critically on the electrode longitudinal distance and position relative to the myotendinous region. Consideration will first be given to the electrophysiological origin of the muscle-shortening effects. Subsequently, we will address all the anatomical and physiological factors that influence the effects of a reduction of the muscle length. Finally, the dependence of the muscle-shortening effects on the electrode longitudinal position will be discussed and interpreted using the above factors.

### The electrophysiological origin of the muscle-shortening effects

**Fig. 10:**
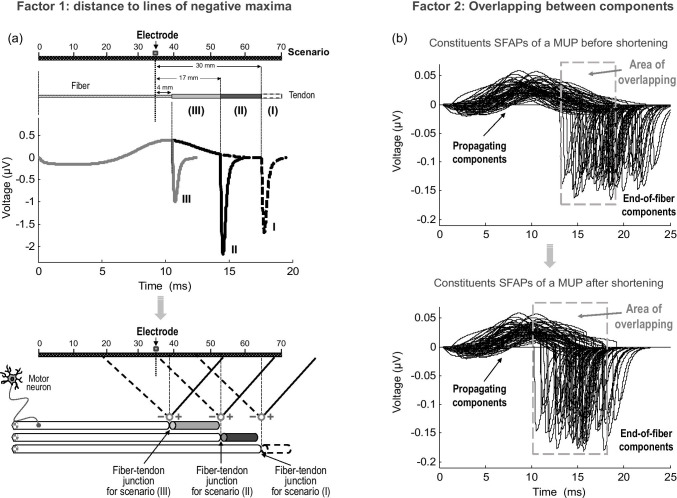
**a** Upper panel—simulation of single-fiber potentials for 3 different scenarios corresponding to different longitudinal distances between the electrode and the fiber-tendon junction: 30 mm, scenario (I); 17 mm, scenario (II); and 4 mm, scenario (III). **a** Bottom panel—schematic representation of the dipole field (in the form of lines of positive and negative maxima) generated by the stationary dipoles lying on the fiber-tendon junction for the three scenarios defined above. **b** Simulation of the constituents single-fiber potentials (SFAPs) of a motor unit potential before (upper panel) and after (lower panel) muscle shortening of 20% for the electrode located at 30 mm from the innervation zone. Note the overlapping between the propagating and non-propagating components of the different SFAPs

Extracellular potentials recorded from skeletal muscles represent the superposition of two signals of different nature: one resulting from the propagation of action potentials along the fiber membrane (the so-called propagating component) and the other arising from the extinction of these potentials at the tendon (non-propagating or end-of-fiber component). Due to their different electrical formation, the propagating and non-propagating components are affected in a different manner by the geometrical changes imposed by fiber shortening. More specifically, the non-propagating component can be seen to arise from a stationary source located at a fixed location, the fiber-tendon junction. Therefore, positional changes of this junction relative to the recording electrode alter the magnitude of the whole *end-of-fiber* component [13]. Conversely, the propagating component results from the propagation of the action potential along the muscle fiber. Thus, a shift of the fiber-tendon junction due to fiber shortening only affects the point at which the propagating component is terminated, but not the magnitude of this component (see Fig. 10a). In conclusion, changes in extracellular potentials resulting from muscle shortening mainly reflect modifications in the *end-of-fiber* components.

### Factors affecting the muscle-shortening effects

There are several factors that play a role in the muscle-shortening effects. At the single-fiber level, there are two factors influencing the amplitude of *end-of-fiber* components. One is the electrode position and distance relative to the fiber-tendon junction, and the other is the distance from the electrode to the lines of positive and negative maxima emerging from the stationary dipole. This second factor, i.e., the proximity to the lines of maxima, dominates over the first one and, as a result, the amplitude of *end-of-fiber* signals varies in a complex way as a function of the longitudinal distance between the electrode and the fiber-tendon junction (see the next subsection). Moreover, at the motor unit and muscle level, there are two additional confounding factors that further complicates the shortening effects. First, the issue of overlapping. Indeed, because the individual constituents of a compound potential are not aligned in time, the propagating component of some fibers overlaps with the non-propagating component of other fibers. Muscle shortening accentuates this overlapping, thereby modifying the interaction between the propagating and non-propagating components. The second complicating factor is the spatial spreading of the fiber-tendon junctions of a motor unit/muscle. This spreading influences the summation of the propagating and non-propagating components, which is different for the resting and shortened muscle. The impact of each of these factors is discussed next.

### Impact of the distance between the electrode and the line of negative and positive maxima

As explained in the methods, *end-of-fiber* components are generated by stationary dipoles that arise at the fiber-tendon junction when the traveling action potential dies at this junction. The electrical field emerging from a stationary dipole does not have spherical symmetry; rather, it has positive and negative maximal points, which are contained within straight lines of known equation (see methods). As a result, the magnitude of end-of-fiber potentials depends not only on the distance from the electrode to the stationary dipole, but also, and especially, on the distance from the electrode to the lines of positive and negative maxima emerging from this dipole. Indeed, this last factor makes the amplitude of *end-of-fiber* signals vary in a counterintuitive way as a function of the longitudinal distance between the electrode and the fiber-tendon junction. To illustrate this complexity, several scenarios are proposed in Fig. 10a. In scenario (I), the electrode is further from the line of negative maxima compared to the electrode of scenario (II) and, as a result, the end-of-fiber potential is smaller in the first scenario. In fact, in scenario (II), the electrode lies on the line of negative maxima, and hence the end-of-fiber potential recorded in this scenario has the largest amplitude along the muscle longitudinal axis. Moreover, the electrode in scenario (III), despite being closer to the stationary dipole, records a potential with smaller amplitude than the electrode in scenario (II). This is because the electrode in scenario (III) is further from the line of negative maxima than is the electrode in (II).

### Impact of the overlapping between the propagating and non-propagating components

The individual constituents of a compound potential are dispersed in time, and hence, irrespective of the electrode position, the propagating component of some fibers inevitably overlaps with the non-propagating component of other fibers (see Fig. 10b). This overlapping occurs for all electrode positions, but is particularly relevant for electrode locations close to the myotendinous region (distances shorter than about 30 mm). The reason is that, for these recording positions, there is a shorter time separation between the positive maximum of the propagating component and the negative maximum of the non-propagating (end-of-fiber) component (see Fig. 10 for an example). With such short time separation, there occurs high overlapping between the main “positive” (propagating) phase of some fibers and the “negative” non-propagating components of other fibers. Interacting, the overlapping phases diminished each other. Muscle shortening enlarges the portion of overlapping, thereby increasing the phase cancelation between the propagating and non-propagating components of the different fibers. Therefore, the net result of the increased overlapping caused by shortening is a decreasing effect on the final “negative” phase of the compound potential and also a decreasing effect on the main “positive” phase, as can be appreciated in Fig. 5(c), Fig. 7(c), and Fig. 8(b, c).

### Shortening effects for composite potentials depending on the electrode longitudinal position

Our findings on the shortening effects for MUPs and M waves can be explained by the possible combinations of the above factors (distance to the lines of negative and positive maxima and overlapping). Four general situations can be distinguished, depending on the electrode longitudinal position. The first scenario corresponds to the electrode being placed far from the myotendinous zone (see Fig. 5(a), *d*_IZ_ = 20 mm). In this case, as a result of muscle shortening, all the lines of negative maxima are brought closer to the electrode, thus producing greater end-of-fiber components, and hence an increase of the MUP final phase. For these electrode locations, overlapping is not a factor, since propagating and non-propagating components are far apart from each other.

A second scenario occurs when the electrode is placed midway between the innervation zone and myotendinous regions (see Fig. 5(b), *d*_IZ_ = 30 mm). For this electrode position, muscle shortening still shifts the lines of negative maxima closer to the electrode, thus leading to greater end-of-fiber components. Additionally, in this scenario, the overlapping between the propagating and non-propagating components of the different fibers is significantly increased due to muscle shortening (see Fig. 10b). Thus, the enhanced phase cancelation caused by overlapping produces a decreasing effect on the MUP final phase, which counteracts the increasing effect due to the reduced distance to the lines of negative maxima. The net result is that the amplitude of the MUP final phase is approximately the same before and after shortening, whereas the amplitude of the MUP main positive phase does not change.

In the third scenario, with the electrode lying in the proximity of the myotendinous zone (see Fig. 5(c), *d*_IZ_ = 40 mm), a shortening of muscle fibers moves most of all the lines of negative maxima further from the electrode, thus producing smaller end-of-fiber components. Besides, for this electrode position, there is a high overlapping between the propagating and non-propagating components of the different fibers. Muscle shortening further accentuates this overlapping, thus increasing phase cancelation. Therefore, both factors (distance to lines of negative maxima and overlapping) act in the same direction to decrease the MUP final phase. Besides, due to the exaggerated overlapping, the main “positive” phase of the MUP is also decreased.

A fourth scenario can be recognized in which the electrode lies beyond the myotendinous zone (see Fig. 5(e, f), *d*_IZ_ = 70, 80 mm). In these conditions, a shortening of muscle fibers move most of all the lines of positive maxima further from the electrode, thus producing smaller (but positive) end-of-fiber components, and hence a decrease of the MUP final (positive) phase.

### The effect of spatial spreading of the myotendinous zone

We found that the spatial dispersion (scattering) of the fiber-tendon junctions of a motor unit/muscle can significantly modify the muscle-shortening effects. The reason is that spreading the ending points of the fibers within a motor unit (or muscle) over a wider area leads to a higher time dispersion between the individual end-of-fiber components of the compound potential, which has important implications. First, the more dispersed end-of-fiber components result in a less effective summation of these components, and hence in a decrease of the amplitude of the MUP (and M-wave) final phase. Second, the more dispersed non-propagating components enlarge the portion of overlapping with the propagating components, thereby increasing the phase cancelation. Finally, it must be noted that, whereas the dispersion between the end-of-fiber components increases due to the myotendinous zone spreading, the timing between the propagating components is not affected by this spreading.

In electrical terms, the difference between motor units with wide and narrow myotendinous zones is that, in the first ones, the propagating components interact with “more dispersed” end-of-fiber components; in other words, the propagating components overlap with “less densely packed” end-of-fiber components. Thus, when the overlapping between these components increases due to muscle shortening, the enhanced phase cancelation results in a greater decreasing effect on the MUP final phase for the wide than for the narrow myotendinous zone. This explains, for example, why, for the electrode at *d*_IZ_ = 20 mm, muscle shortening caused an enlargement of the MUP final phase for a motor unit with a narrow myotendinous zone (Fig. 7(a)), but not for one with a narrow myotendinous zone (Fig. 7(e)).

In a composite potential, the overlapping between the propagating and non-propagating components increases as the distance to the myotendinous zone decreases. It has been shown that, if this distance is sufficiently short, the increase in the overlapping provoked by muscle shortening results in a decrease of the main propagating phase of the MUP (Fig. 5(c)). Then, because a wider myotendinous zone enlarges the portion of overlapping between the propagating and non-propagating components, the decreasing effect of muscle shortening on the main positive phase of the MUP starts occurring at further distances from the myotendinous zone. This explains why, for a narrow spreading of the fiber endings, the amplitude of the MUP positive phase is decreased by muscle shortening at *d*_IZ_ = 40 mm (Fig. 7(c)), whereas, for a wide spreading, the decrease is observed even at *d*_IZ_ = 30 mm (Fig. 7(f)) (i.e., at a much further distance from the myotendinous zone).

### Practical applications and implications

There are several mechanisms/scenarios that can induce a shortening of the muscle fibers which could have important effects on SFAPs, MUPs, and M waves. First, muscle shortening during sustained isometric contractions is due to motor unit recruitment and, indeed, muscle fiber length decreases progressively throughout the contraction as motor units are progressively recruited. In support of this, it has been shown that during a sustained isometric contraction, the fascicle length decreases and its pennation angle increases, these changes being more marked during the initial part of the contraction [22]. A second factor that induces muscle shortening is a decrease in tendon stiffness. Such reduction in tendon stiffness could occur, for example, after prolonged high-intensity contractions [20]. Importantly, a reduced tendon stiffness would cause the muscle to operate at a shorter length, a factor that has been shown to increase the amplitude of the M-wave second phase, as demonstrated in the present manuscript. A third scenario where muscle shortening is likely to occur is after a brief (< 10 s) isometric contraction. Specifically, it has been proposed that muscle fiber length may remain shortened for a few seconds (~ 15 s) after a brief isometric contraction [35]. It is this transient shortening of muscle fibers the mechanism that would explain the transient increase in the amplitude of the M-wave second phase and the decrease in M-wave duration after a brief voluntary contraction [34]. The fourth scenario where muscle shortening occurs is when contracting the muscle at different joint angles: in this case, the muscle length at rest (passive condition) varies depending on the joint angle.

Finally, a clarification must be made as some authors have suggested that the main effect of muscle shortening on extracellular EMG potentials is due to the increase in conduction velocity [4, 7]. However, in the present study, it has been demonstrated that, even in the absence of changes in conduction velocity, muscle shortening significantly alters the shape of individual SFAPs, MUPs, and M waves.

## Conclusion

In conclusion, it has been shown that muscle shortening has complex effects on EMG potentials and that these effects essentially reflect modifications in the *end-of-fiber* components of the potential and the way they interact with the propagating components. At the single-fiber level, two main factors influence the muscle-shortening effects: (1) the electrode position and distance relative to the fiber-tendon junction and (2) the electrode distance to the lines of positive and negative maxima emerging from the stationary dipoles at the fiber-tendon junctions. Besides, at the motor unit and muscle level, two additional factors are involved: (3) the overlapping between the propagating component of some fibers with the non-propagating component of other fibers and (4) the spatial spreading of the fiber-tendon junctions. The dependence of the muscle-shortening effects on the electrode longitudinal position can be roughly described as follows. When the electrode is placed far from the myotendinous zone, muscle shortening resulted in an enlargement and narrowing of the final (negative) phase of the potential, and this enlargement becomes less and less pronounced as the electrode approaches the fiber endings. For electrode locations close to the myotendinous zone, muscle shortening causes a decrease in the amplitude of both the main (positive) and final (negative) phases of the potential. Beyond the myotendinous zone, muscle shortening leads to a decrease of the final (positive) phase. The present results provide reference information that will help to identify changes in MUPs and M waves due to muscle shortening, and thus to differentiate these changes from those caused by physiological variables.
